# Using the dCas9-KRAB system to repress gene expression in hiPSC-derived *NGN2* neurons

**DOI:** 10.1016/j.xpro.2021.100580

**Published:** 2021-06-03

**Authors:** Aiqun Li, Samuel Cartwright, Alex Yu, Seok-Man Ho, Nadine Schrode, P.J. Michael Deans, Marliette R. Matos, Meilin Fernandez Garcia, Kayla G. Townsley, Bin Zhang, Kristen J. Brennand

**Affiliations:** 1 Department of Genetics and Genomic Sciences, Icahn School of Medicine at Mount Sinai, One Gustave L. Levy Place, New York, NY 10029, USA; 2 Mount Sinai Center for Transformative Disease Modeling, Icahn School of Medicine at Mount Sinai, One Gustave L. Levy Place, New York, NY 10029, USA; 3 Icahn Institute for Data Science and Genomic Technology, Icahn School of Medicine at Mount Sinai, One Gustave L. Levy Place, New York, NY 10029, USA; 4 Nash Family Department of Neuroscience, Icahn School of Medicine at Mount Sinai, 1425 Madison Avenue, New York, NY 10029, USA; 5 Friedman Brain Institute, Icahn School of Medicine at Mount Sinai, 1425 Madison Avenue, New York, NY 10029, USA; 6 Department of Psychiatry, Icahn School of Medicine at Mount Sinai, 1425 Madison Avenue, New York, NY 10029, USA

**Keywords:** Molecular Biology, CRISPR, Neuroscience, Stem Cells, Cell Differentiation

## Abstract

We describe a CRISPR inhibition (CRISPRi) protocol to repress endogenous gene expression (e.g., *ATP6V1A*) in human induced pluripotent stem cell-derived *NGN2*-induced glutamatergic neurons. CRISPRi enables efficient and precise gene repression of one or multiple target genes via delivering gRNA(s) to direct a dCas9-KRAB fusion protein to the gene(s) of interest. This protocol can also be adapted for gene activation and high-throughput gene manipulation, allowing assessment of the transcriptomic and phenotypic impact of candidate gene(s) associated with neurodevelopment or brain disease.

For complete details on the use and execution of this protocol, please refer to Ho et al. (2017) and Wang et al. (2021).

## Highlights

• CRISPRi enables efficient and precise gene repression of one or multiple target genes
• This protocol describes gene repression in hiPSC-derived *NGN2* neurons
• This protocol can be adapted for gene activation and high-throughput gene manipulation

## Graphical Abstract

**Figure:**
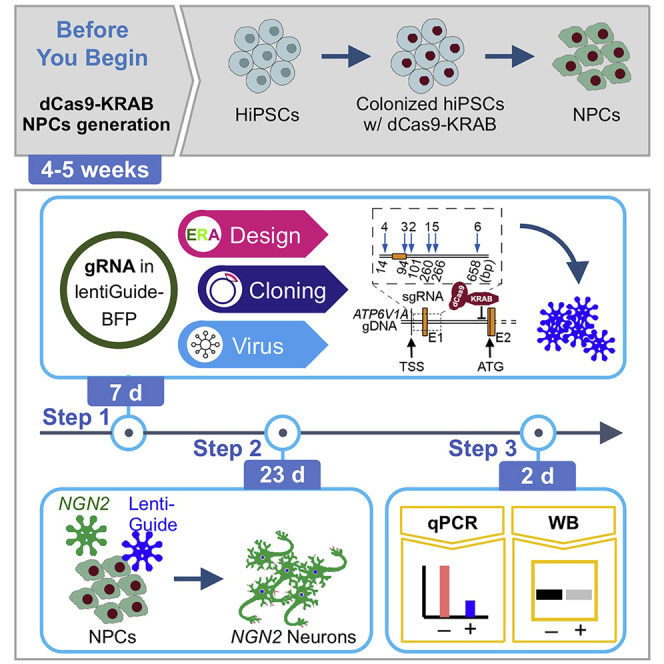

## Before you begin

**Timing: 4–5 weeks**

This protocol uses lentiviral delivery to transduce the stable neural progenitor cells (NPCs) lines expressing the CRISPR-repressor system from human induced pluripotent stem cells (hiPSCs) with pLV-TetO-*hNGN2*-eGFP-Neo, rtTA, and gRNAs. Human *NGN2* (*hNGN2*) can rapidly yield excitatory glutamatergic neurons from NPCs within 21 days. A CRISPR-repressor complex, paired with designed specific gRNA, can repress the expression of the gene of interest. For instance, we utilized CRISPR inhibition (CRISPRi) to reduce endogenous *ATP6V1A*, an example gene in *NGN2* neurons (Wang et al., 2021). Researchers can manipulate their gene(s) of interest using a similar approach.

### Generation of dCas9-KRAB NPCs from hiPSCs

Human neurons for CRISPRi can be induced from hiPSCs (Zhang et al., 2013) or NPCs (Ho et al., 2016**)**. There are distinct advantages to either starting cell: hiPSCs can be clonally manipulated and expanded indefinitely, while NPCs are easily transduced and already patterned towards a neural identity. Here we describe methods to apply CRISPRi specifically to NPC-induced *NGN2* glutamatergic neurons. The inhibitory action performed by CRISPRi is allowed with the use of the Kruppel-associated box (KRAB) domain fused to dead Cas9 (dCas9-KRAB), which can repress gene transcription efficiently (Gilbert et al., 2013). Towards this, the generation of NPCs that stably express dCas9-KRAB is a timesaving prerequisite for neuronal differentiation and gene repression. Please see our previous publications for details (Ho et al., 2016; Topol et al., 2015). Here is a brief description of how to first generate NPCs from hiPSCs and second, how to engineer them to overexpress dCas9-KRAB.

1. Generation of NPCs from hiPSCs.
  HiPSC-NPCs are generated as previously described in detail (Topol et al., 2015).
  a. Differentiate hiPSCs into embryoid bodies (EBs) in suspension culture (DMEM/F12 and 10% fetal bovine serum (FBS)), with dual-SMAD inhibition (0.1 μM LDN193189 and 10 μM SB431542) to improve yield, as described (Topol et al., 2015) (∼7 days).
  b. Plate and further differentiate EBs until neural rosettes appear (∼14 days).
  c. Manually pick neural rosettes and dissociate to generate NPCs in NPC medium (see materials and equipment) on Matrigel (BD, #354277). HiPSC-NPCs, at full confluence (1–1.5 × 10^7^ cells/well of a 6-well plate), are dissociated with Accutase (Innovative Cell Technologies, #AT104) for 5 min, spun down (5 min, 1,000 × *g*), resuspended, and seeded onto Matrigel-coated plates at a cell density of 1.5–2.5 × 10^6^ cells/mL. Change media every 2 days for 4–7 days until next split.
  d. If necessary, NPCs can be further purified by MACS-based purification to enrich for CD271–/CD133+ cells (Miltenyi Biotech #130-097-127 and #130-091-895) (Bowles et al., 2019).
2. Generation of antibiotic-selected dCas9-KRAB NPCs (Ho et al., 2017).
  a. Seed 3.0 × 10^6^ NPCs per well into Matrigel-coated 6-well plates in 2.0 mL NPC medium.
  b. Next day, use the lentivirus of lenti-EF1a-dCas9-KRAB-Puro (Addgene Plasmid #99372) for spinfection (1 h, 1,000 × *g*, 25°C, Ho et al., 2016).
  c. Following spinfection, plates are transferred to an incubator for 3–4 h. Exchange with fresh NPC medium.
  d. After 2 days, exchange media. Add fresh NPC medium containing 1 μg/mL puromycin.
  e. Expanded cells in NPC medium containing 1 μg/mL puromycin. Banking in liquid nitrogen. Each vial contains 1 × 10^6^ viable NPCs.
  f. Once thawed, grow NPCs in NPC medium containing 1 μg/mL puromycin.

## Key resources table

**Table:** 

REAGENT or RESOURCE	SOURCE	IDENTIFIER
**Antibodies**		

Recombinant Anti-ATP6V1A antibody (1:1,000) (stored at 4°C for 1–2 weeks, –20°C or –80°C for long term)	Abcam	Cat# ab199326, RRID:AB_2802119
Anti-MAP2 antibody (1:2,000) (stored at 4°C for 1–2 weeks, –20°C or –80°C for long term)	Abcam	Cat# ab5392
Mouse monoclonal anti-β-Actin antibody (1:1,000) (stored at 4°C for 1–2 weeks, –20°C or –80°C for long term)	Abcam	Cat# ab8227, RRID: AB_2305186
Purified anti-Tubulin β 3 (TUBB3)/TUJ1 Antibody (stored at 4°C for 1–2 weeks, –20°C or –80°C for long term)	BioLegend	Cat# 801202, RRID:AB_10063408
IRDye 680RD Donkey anti-Rabbit IgG (1:15,000) (store at 4°C for up to 3 months)	LI-COR Biosciences	P/N 925-68073: RRID AB_2716687
IRDye 800CW Donkey anti-Mouse IgG (H + L) (1:15,000) (store at 4°C for up to 3 months)	LI-COR Biosciences	P/N 925-32212: RRID AB_2716622

**Bacterial and virus strains**		

NEB 10-beta Competent E. coli (stored at –80°C)	New England Biolabs	Cat# C3019H
Human embryonic kidney (HEK) 293T cells (stored in liquid nitrogen freezers)	Invitrogen	Cat# R700-07

**Chemicals, peptides, and recombinant proteins**		

Accutase (stored at –20°C)	Innovative Cell Technologies	Cat# AT104
abm’s Lentivirus Titration Kit standard 1 (STD 1) (stored at –20°C)	abm	Part# LV900-B
abm’s Lentivirus Titration Kit standard 2 (STD 2) (stored at –20°C)	abm	Part# LV900-B
Agar powder (pure) (stored at 15°C–30°C)	Alfa Aesar	Cat# A10752
Ampicillin (stored at –20°C)	Sigma	Cat# A9518
ATP (10 mM) (stored at –20°C)	New England Biolabs	Cat# P0756S
B-27 Supplement (50×), minus vitamin A (stored at –80°C)	Life Technologies	Cat# 12587010
BDNF (rh/m/r/c/e) - lyophilized - 25 μg (stored at –80°C)	R&D Systems (R&D)	Cat# 248-BD-025
BrainPhys basal medium (stored at 4°C)	STEMCELL Technologies	Cat# 5790
BsmBI (10,000 U/mL) (stored at –20°C)	New England Biolabs	Cat# R0739S
Cytosine-β-D-arabinofuranoside hydrochloride (Ara-C) (stored at –20°C or –80°C)	Sigma	Cat# C1768
Dibutyryl cyclic-AMP (500 mg/mL) (stored at –80°C)	Sigma	Cat# D0627-1G
Difco LB Broth, Miller (Luria-Bertani) (stored at 15°C–30°C)	Fisher Scientific	Cat# DF0446-07-5
DMEM/F-12, GlutaMAX supplement (stored at 4°C)	STEMCELL Technologies	Cat# 10565042
Doxycycline (stored at –20°C)	Sigma	Cat# D9891
FGF basic (rh) – lypholized – 1mg (stored at –80°C)	R&D Systems (R&D)	Cat# 233-FB-01M
G418 (Geneticin) (stored at –20°C)	Sigma	Cat# 11811031
GDNF (rh) - 50 μg (stored at –80°C)	R&D Systems (R&D)	Cat# 212-GD-050
Halt Protease and Phosphatase Inhibitor Cocktail	Thermo Fisher Scientific	Cat# 78440
Hygromycin (stored at –20°C)	Sigma	Cat# 10687010
L-Ascorbic acid (200 mM) (stored at –80°C)	Sigma	Cat# A0278
Laminin Mouse Protein, Natural (1 mg/mL) (stored at –80°C)	Life Technologies	Cat# 23017015
N-2 supplement (100×) (stored at –80°C)	Life Technologies	Cat# 17502048
NP-40 (10%) (stored at 20°C–25°C)	Thermo Fisher Scientific	Cat# 85124
Opti-MEM I Reduced Serum Medium, no phenol red (stored at 4°C)	Life Technologies	Cat# 11058021
PEI (stored at 15°C–30°C)	Polysciences, Inc.	Cat# 23966-2
Phosphate-buffered saline (PBS) (stored at 15°C–30°C)	Life Technologies	Cat# 10010031
Puromycin (stored at –20°C)	Sigma	Cat# P7255
RIPA Lysis and Extraction Buffer (stored at 4°C)	Thermo Fisher Scientific	Cat# 89900
S.O.C. Medium (stored at 15°C–30°C)	Invitrogen	Cat# 15544034
Sodium deoxycholate (10%) (stored at 15°C–30°C)	Fisher Scientific	Cat# 50-255-883
SDS (10%) (stored at 15°C–30°C)	Thermo Fisher Scientific	Cat# 15553027
T4 Polynucleotide Kinase (T4 PNK) (10,000 U/mL) (stored at –20°C)	New England Biolabs	Cat# M0201S
T4 Polynucleotide Kinase Reaction Buffer (10× PNK buffer) (stored at –20°C)	New England Biolabs	Cat# B0201S
Terrific Broth (stored at 15°C–30°C)	Fisher Scientific	Cat# BP2468-2
UltraPure DNase/RNase-Free Distilled Water (stored at 15°C–30°C)	Life Technologies	Cat# 10977015

**Critical commercial assays**		

abm’s qPCR Lentivirus Titration Kit (includes Virus Lysis Buffer) (stored at –80°C)	abm	Cat# LV900
BSA (20 ng/μL) (stored at –20°C)	Invitrogen	Cat# 15561020
ECL Prime Western Blotting Detection Reagents	GE Healthcare	Cat# RPN2236
GeneJET Plasmid Miniprep Kit (stored at 15°C–30°C)	Thermo Scientific	Cat# K0502
Multi-electrode array (MEA)	Axion BioSystems	Cat# M768-MEA-48W
Power SYBR Green RNA-to-Ct 1-Step Kit (stored at –20°C)	Thermo Fisher Scientific	Cat# 4389986
QIAprep Spin Miniprep Kit (stored at 15°C–30°C)	QIAGEN	Cat# 27104
Quick Ligase Buffer (2×) (stored at –20°C)	New England Biolabs	Cat# M2200S
Quick Start Bradford Protein Assay Kit 1 (store at 4°C)	Bio-Rad	Cat# 5000201
SYBR Select Master Mix (stored at –20°C)	Applied Biosystems	Cat# 4472903
T7 DNA Ligase (3,000,000 U/mL) (stored at –20°C)	New England Biolabs	Cat# M0318S
TaqMan RNA-to-Ct 1-Step Kit (stored at –20°C)	Invitrogen	Cat# 4392938
TRIzol Reagent (stored at 4°C)	Thermo Fisher Scientific	Cat# 15596018

**Deposited data**		

*ATP6V1A* knock-down RNA sequencing data	(Wang et al., 2021)	Gene expression omnibus (GEO): GSE128367

**Experimental models: Cell lines**		

Two stable hiPSC-derived neuronal progenitor cells (hiPSC-NPCs) expressing dCas9^-KRAB^ (Addgene plasmid #99372)	Brennand Lab at Icahn School of Medicine at Mount Sinai	553-S1-1 KRAB and 2607-1-4 KRAB
Human astrocytes	ScienCell	Cat# 1800
Human embryonic kidney (HEK) 293T cells	ATCC	Cat# CRL-3216

**Oligonucleotides**		

gRNA sequences to repress *ATP6V1A* expression: i_1 seq: FWD: 5’-**CACCG***GCGGGAACGACCACACTTGG*-3’ i_2 seq: FWD: 5’-**CACCG***GGCGACCGGTAACTGGCGAG*-3’	Wang et al., 2021	N/A
qPCR primers for *ATP6V1A* FWD*:* 5’-GAGATCCTGTACTTCGCACTG-3’ REV: 5’- GGGATGTAGATGCTTTGGGTC-3’	Wang et al., 2021	N/A
qPCR primers for β-*Actin* FWD: 5’-TGTCCCCCAACTTGAGATGT-3’ REV: 5’-TGTGCACTTTTATTCAACTGGTC-3’	Wang et al., 2021	N/A
U6 primer	Guo et al., 2012	Addgene Plasmid #40644

**Recombinant DNA**		

lentiGuide-Hygro-mTagBFP2 (stored at –80°C)	Ho et al., 2017	Addgene Plasmid #99374
lenti-EF1a-dCas9-KRAB-Puro (stored at –80°C)	Ho et al., 2017	Addgene Plasmid #99372
FUW-M2rtTA (stored at –80°C)	Hockemeyer et al., 2008	Addgene Plasmid #20342
pLV-TetO-h*NGN*2-eGFP-Neo (stored at –80°C)	Wang et al., 2021	N/A
pMDLg/pRRE (MDL) (stored at –80°C)	Dull et al., 1998	Addgene Plasmid #12251
pRSV-Rev (Rev) (stored at –80°C)	Dull et al., 1998	Addgene Plasmid #12253
pCMV-VSV-G (VSVG) (stored at –80°C)	Stewart et al., 2003	Addgene Plasmid #8454

**Software and algorithms**		

SnapGene Viewer	GSL Biotech LLC	snapgene.com
Prism 7	GraphPad	GraphPad.com
CRISPR-ERA (sgRNA Design Tool)	Liu et al., 2015	crispr-era.stanford.edu

**Other**		

AB Veriti 96-well Thermal Cycler	Applied Biosystems	Cat# 4375786
Countess 3 Automated Cell Counter	Invitrogen	Cat# AMQAX2000
Eppendorf Centrifuge 5702R	Eppendorf	Cat# 022626205
Eppendorf Model 5810R Centrifuge	Eppendorf	Cat# 022625501
LI-COR Odyssey Classic Imaging System	LI-COR Biosciences	Model# 9120
Olympus IX51 Microscope	Olympus	N/A
Optima L-100XP Ultracentrifuge	Beckman Coulter	Cat# 392052
Optima XE-90 with 32 SW-Ti rotor	Beckman Coulter	Cat# A94471
Polypropylene centrifuge tubes	Beckman Coulter	Cat# 326823
StepOnePlus Real-Time PCR Machine	Applied Biosystems	Cat# 4376600
Ultrasonic cleaner	Grainger	Cat# 32V119

## Materials and equipment

**Table:** 

Antibiotics stocks	Final concentration	Amount
Hygromycin	1 mg/mL	500 μL/vial
G418 (Geneticin)	1 mg/mL	500 μL/vial
Puromycin	1 μg/mL	500 μL/vial

Can be stored at –20°C for several months.

**Table:** 

NPC medium	Final concentration	Amount
DMEM/F-12, GlutaMAX	-	48.425 mL
100× N-2 Supplement	1×	0.5 mL
50× B-27 Supplement, minus vitamin A	1×	1 mL
FGF-Basic (rh) (20 μg/mL)	20 ng/mL	0.05 mL
Laminin Mouse Protein, Natural (1 mg/mL)	0.5 μg/mL	0.05 mL
**Total**		**50 mL**

Can be stored at 4°C for one month.

**Table:** 

Neuron medium	Final concentration	Amount
BrainPhys basal medium	-	48.275 mL
100× N-2 Supplement	1×	0.5 mL
50× B-27 Supplement, minus vitamin A	1×	1 mL
Laminin Mouse Protein, Natural (1 mg/mL)	0.5 μg/mL	25 μL
BDNF (20 μg/mL)	20 ng/mL	50 μL
GDNF (20 μg/mL)	20 ng/mL	50 μL
Dibutyryl cyclic-AMP (500 mg/mL)	500 μg/mL	50 μL
L-Ascorbic acid (200 mM)	200 μM	50 μL
**Total**		**50 mL**

Can be stored at 4°C for one month.

**Table:** 

Agar plates	Final concentration	Amount
Agar powder (pure)	2 mg/mL	1.0 g
Lysogeny Broth (LB)	25 mg/mL	12.5 g
DNase/RNase-free distilled water		500 mL
Ampicillin	100 μg/mL	500 μL

Can be stored at 4°C for two months.

**Table:** 

Alternative RIPA Lysis Buffer	Final concentration	Amount
NaCl (5 M)	150 mM	3 mL
Tris-HCl (1 M)	50 mM	5 mL
NP-40 (10%)	1%	10 mL
Sodium deoxycholate (10%)	0.5%	5 mL
SDS (10%)	0.1%	1 mL
DNase/RNase-free distilled water		76 mL
**Total**		**100 mL**

Can be stored at –20°C for several months.

## Step-by-step method details

### Guide RNA design to target a gene of interest

**Timing: 0.5–1 day**

**Figure 1:**
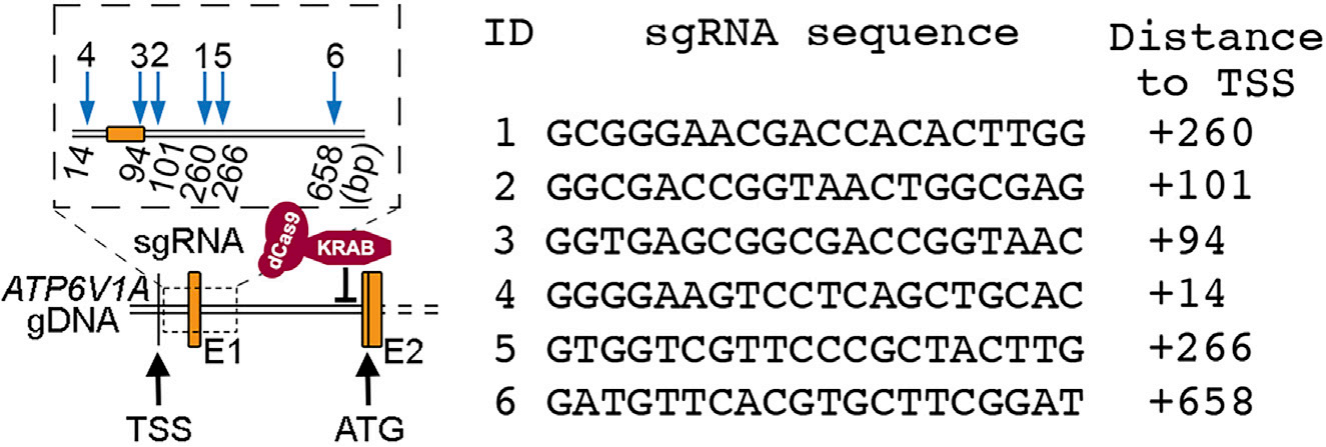
Example of CRISPR-ERA generated sgRNAs to target the promoter region for *ATP6V1A* knockdown (KD) E1: exon 1; E2: exon 2; TSS: transcription start site; ATG is the translation initiation codon.
3. Create a manifest file containing information with target gene name, species, distance to TSS, E (efficacy) + S (Specificity) scores, sgRNA sequence, and CRISPR application.
  a. Note that the sgRNA sequences should be sure to include the oligo overhangs. Importantly, we add a ‘G’ as the initiation nucleotide, making the transcription more efficient.
  b. Our lab usually adds a ‘G’ when ordering oligos. On the 5’ end of the Forward Oligo, there will be a ‘CACCG’ sequence, and on the 5’ end of the Reverse Oligo, there will be an ‘AAAC’ sequence. The overhangs are required for the oligos to be complementary to the linearized plasmid backbone during ligation, which will allow for successful cloning. Thus, the oligo orders should be ‘5’-CACCGN...N-3’ and ‘5’- AAACN...NC-3’.
  c. The ordered sgRNAs will arrive as two single-stranded guide RNA per gRNA. An annealing step must be performed prior to cloning.
4. (Optional. This step is additional verification for efficiency and specificity of designed sgRNAs.) Off-target sgRNA comparison prevents unwanted gene manipulation.
  a. To check for off-target interactions for your generated sgRNAs you can use various tools, we used http://crispor.tefor.net/.
  b. For ‘step 1’ copy and paste the binding element sequence for the sgRNA you wish to verify.
    i. The sequence must be at least 20 base pairs long.
  c. Second, for ‘step 2’, select the genome of interest.
    i. We chose ‘Homo sapiens – Human – UCSC Feb. 2009 (GRCh37/hg19) + SNPs: 1000Genomes, ExaC’.
  d. Third, for ‘step 3’, select the Protospacer Adjacent Motif (PAM).
    i. We used the default selection ‘20bp-NGG-Sp Cas9, SpCas9HF1, eSpCas9 1.1’.
  e. Select ‘Submit’, and wait about 10–15 s to get the results.
  f. The two specificity scores, MIT and CFD, can then be used to gauge the likelihood of your sgRNA having off-target genome effects.
    i. The scores range from 0–100, with the higher scores indicating a lower off-target effect on the genome of interest.
    ii. sgRNAs should have a specificity score greater than 50 to be utilized for cloning and later transfection.

***Note:*** The U6 RNA Pol III promoter transcription starts at the +1 position (23 nucleotides after the TATA box), with G as the preferred initiation nucleotide for RNA Pol III promoters. A Guanine nucleotide must be included on one of the 5’ ends of the sgRNA, if it is not already included, to ensure that there is complete and efficient transcription (Kim et al., 2020**)**.

***Note:*** Check whether sgRNAs target the sharing motifs, which may potentially influence the expression of neighboring genes.

1. Search for the gene expression profile in available sequence databases to confirm expression of candidate genes in hiPSC-derived *NGN2-*induced glutamatergic neurons.
  a. Option 1: https://schroden.shinyapps.io/BrennandLab-ExpressionApp/ developed by Dr. Kristen Brennand’s Laboratory, Department of Genetics and Genomic Sciences, Icahn School of Medicine at Mount Sinai, New York.
  b. Option 2: https://ineuronrnaseq.shinyapps.io/rnaseq_app/, created by Connor Ludwig, Kampmann Lab, University of California, San Francisco.
2. Apply a user-friendly web server (http://CRISPR-ERA.stanford.edu) for sgRNAs design.
  a. Select ‘Gene repression’ for step 1 to specify the type of manipulation on the genome.
  b. Select ‘Human(GRCh37/h19)’ for step 2 to specify the organism.
  c. Click ‘Submit step 2, Next Step’.
  d. Type the gene name for step 3 to specify the gene of interest.
  e. Click ‘CRISPR-ERA search’.
  f. Select ‘Using U6 promoter’, as we will clone gRNAs into lentiGuide-Hygro-mTagBFP2 (Addgene, #99374), which contains a U6 promoter.
  g. Select top 6 sgRNAs with highest E+S score that are also well-located within 1 kb upstream from Transcription Start Site (TSS).
    i. If the ‘Distance to TSS’ values of chosen sgRNAs are close (≤ 20 difference) then choose another sgRNA that has the next best E+S score but a greater difference in ‘Distance to TSS’ value to other sgRNAs chosen.
  h. Example of *ATP6V1A* sgRNA selection to repress gene expression: Design 6 sgRNAs to target the promoter region for *ATP6V1A* knockdown (KD) (Figure 1).

### Cloning custom sgRNAs into lentiviral vector of lentiGuide-Hygro-mTagBFP2

**Timing: 1 week**

5. Anneal DNA Oligos
  a. Order 25 nmole DNA oligos.
  b. Reconstitute the dehydrated oligos with UltraPure DNase/RNase-free distilled water to a 100 μM stock. Store at –20°C.
    i. Alternatively, order the oligos in a reconstituted form to save time if working with a large quantity of sgRNA.
  c. Set up the oligo phosphorylation and annealing for each forward (FWD) and reverse (REV) oligos. Oligos can be ordered phosphorylated.

**Table:** 

Reagent	Final concentration	Amount
DNase/RNase-free distilled water	-	5.5 μL
10× PNK buffer	1×	1 μL
ATP (10 mM)	1 mM	1 μL
T4 PNK (10,000 U/mL)	500 U/mL	0.5 μL
**Master Mix Total**		8 μL
FWD Oligo (100 μM)	10 μM	1 μL
REV Oligo (100 μM)	10 μM	1 μL
**Reaction Total**		10 μL
  d. Incubate the reaction mixture in a thermocycler using the following parameters (Figure 2). Following this step, the oligos will be annealed and phosphorylated.

**Figure 2:**
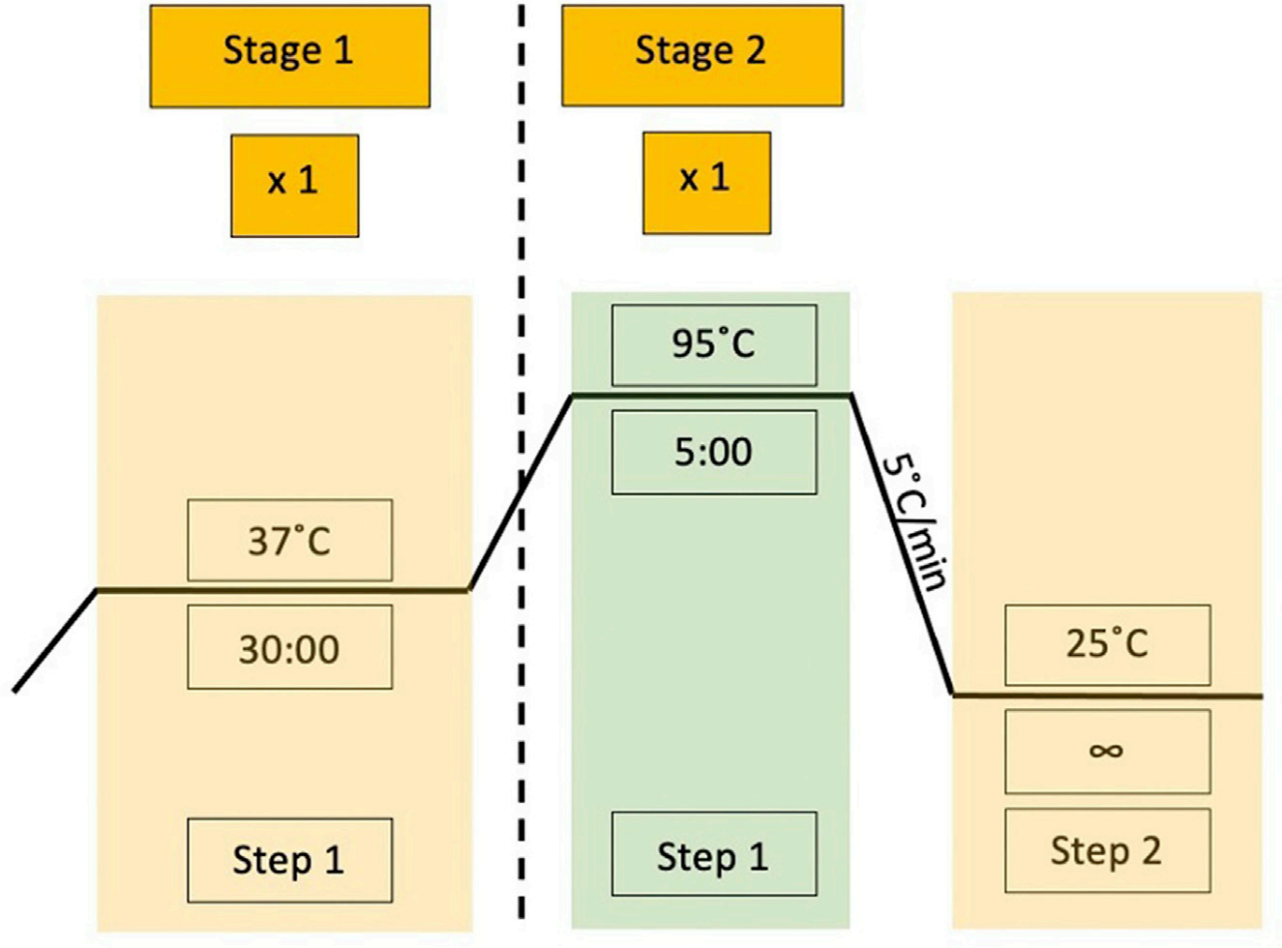
Thermocycler program for annealing
  e. Combine 3 μL of each annealed oligos to create a pool of all annealed oligos. Once pooled, dilute the annealed oligos at a 1:10 dilution with UltraPure DNase/RNase-free distilled water.
    **Pause point:** Store both the un-pooled annealed oligos and the pooled/diluted annealed oligos at –20°C until future use.

**Figure 3:**
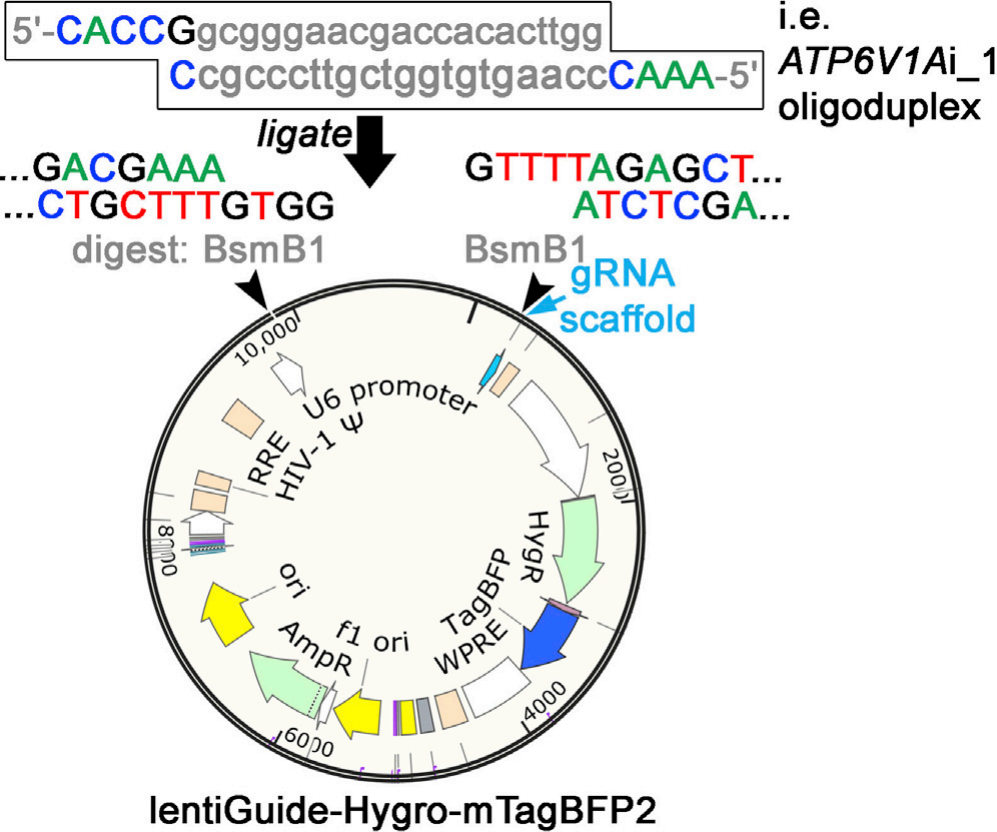
An illustration of BsmBI digestion and ligation to clone an annealed oligos into lentiGuide-Hygro-mTagBFP2 For example, the 20-nucleotide (nt) sequence of *ATP6V1A*i_1 gRNA (lowercase letters in gray) targeting the *ATP6V1A* promoter is cloned into a lentiGuide vector.
  c. Complete the following reaction in a PCR tube.

6. Golden Gate Assembly allows for simultaneous digestion and ligation.
  a. Prepare the backbone of lentiGuide-Hygro-mTagBFP2 with a concentration of 25 ng/μL. Check for the BsmBI recognition sequence prior to running the assembly.
  b. Clone the annealed oligos (from step 5) into the lentiGuide-Hygro-mTagBFP2 plasmid via the BsmBI restriction sites (Figure 3).

**Table:** 

Reagent	Final concentration	Amount
DNase/RNase-free distilled water	N/A	10.25 μL
lentiGuide-Hygro-mTagBFP2	1 ng/μL	1 μL
2× Quick Ligase Buffer	1×	12.5 μL
BSA (20 ng/μL)	0.1 ng/μL	0.125 μL
T7 DNA Ligase (3,000,000 U/mL)	15 U/μL	0.125 μL
BsmBI (10,000 U/mL)	0.4 U/μL	1 μL
**Master Mix Total**		24 μL
Annealed oligos (1:10 diluted)		1 μL
**Reaction Total**		25 μL
  d. Incubate the reaction mixture in a thermal cycler using the following parameters. 15 cycles for Stage 1 (step 1: 5 min at 37°C; step 2: 5 min at 23°C) (Figure 4). 
    **Pause point:** Storage at 4°C for short-term or at –20°C for long-term storage.

**Figure 4:**
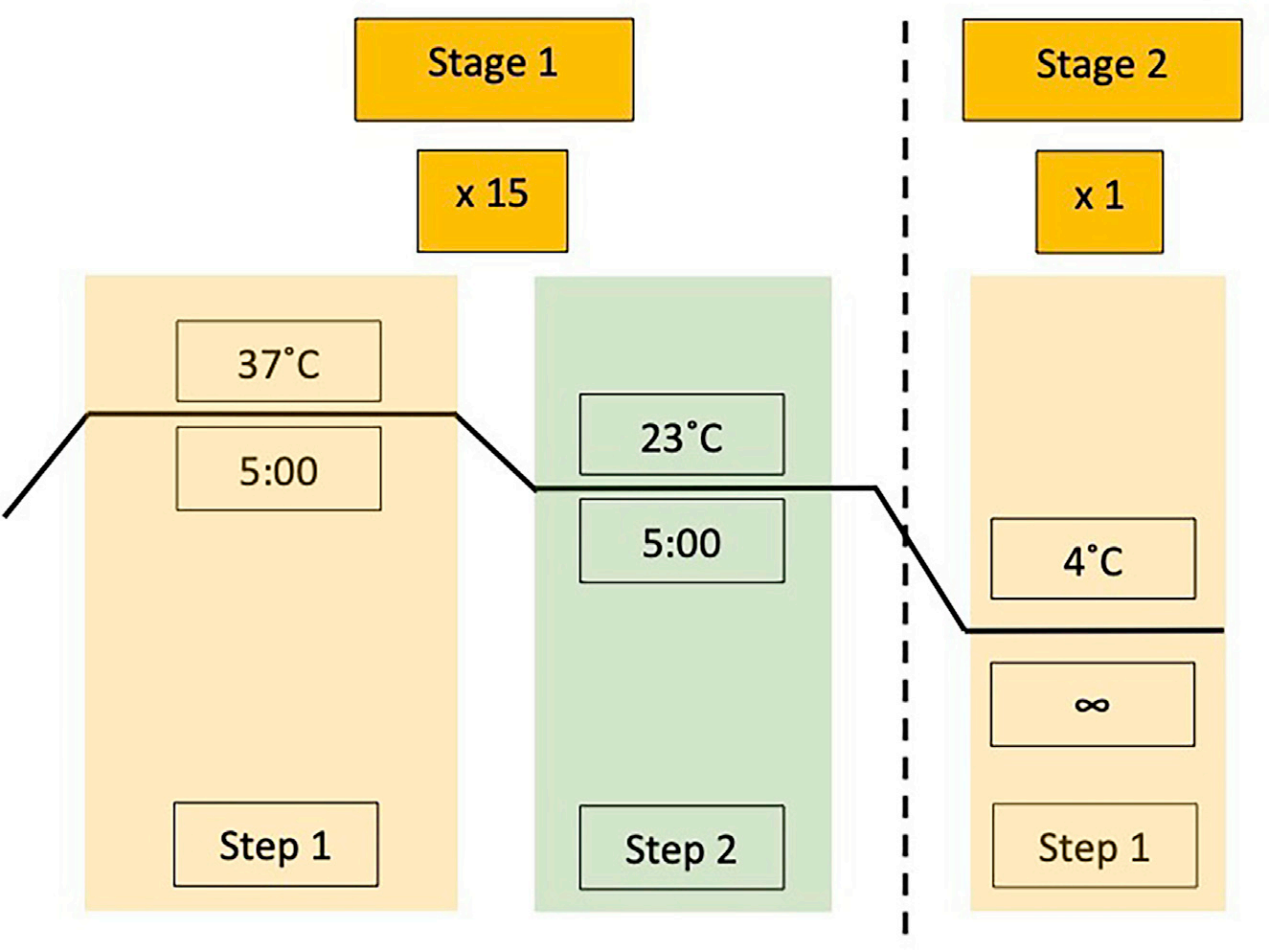
Thermocycler program for digestion and ligation

7. Bacterial transformation & selection
  a. Thaw NEB 10-beta Competent E. coli on ice (approximately 20–30 min).
  b. Remove agar plates (containing the appropriate antibiotic, in this case 100 μg/mL ampicillin) from storage at 4°C, warm up to room temperature (20°C–25°C), and then incubate in 37°C incubator.
  c. Mix 1-5 μL (usually use 1 μL) of DNA (10 pg–100 ng) into 20–50 μL (usually use 20 μL) of competent cells in a microcentrifuge or falcon tube. GENTLY mix by flicking the bottom of the tube with your finger a few times.
  d. Incubate the competent cell/DNA mixture on ice for 20–30 min.
  e. Heat-shock each transformation tube by placing the bottom 1/2 to 2/3 of the tube into a 42°C water bath for 30–60 s (45 sec is usually ideal).
  f. Put the tubes back on ice for 2 min.
  g. Add 250–1,000 μL LB or SOC medium (without antibiotic) to the bacteria and grow in 37°C shaking incubator for 45 min.
  h. Plate some or all of the transformation onto a 10-cm LB agar plate containing the appropriate antibiotic (ampicillin).
  i. Incubate plates at 37°C overnight (18–24 h).
  j. Next morning, check for colonies (see troubleshooting 1).

**Pause point:** Bacterial colony plates may be stored at 4°C for weeks. However, we recommend the verification of recombinant colonies by DNA sequencing soon.

**Figure 5:**
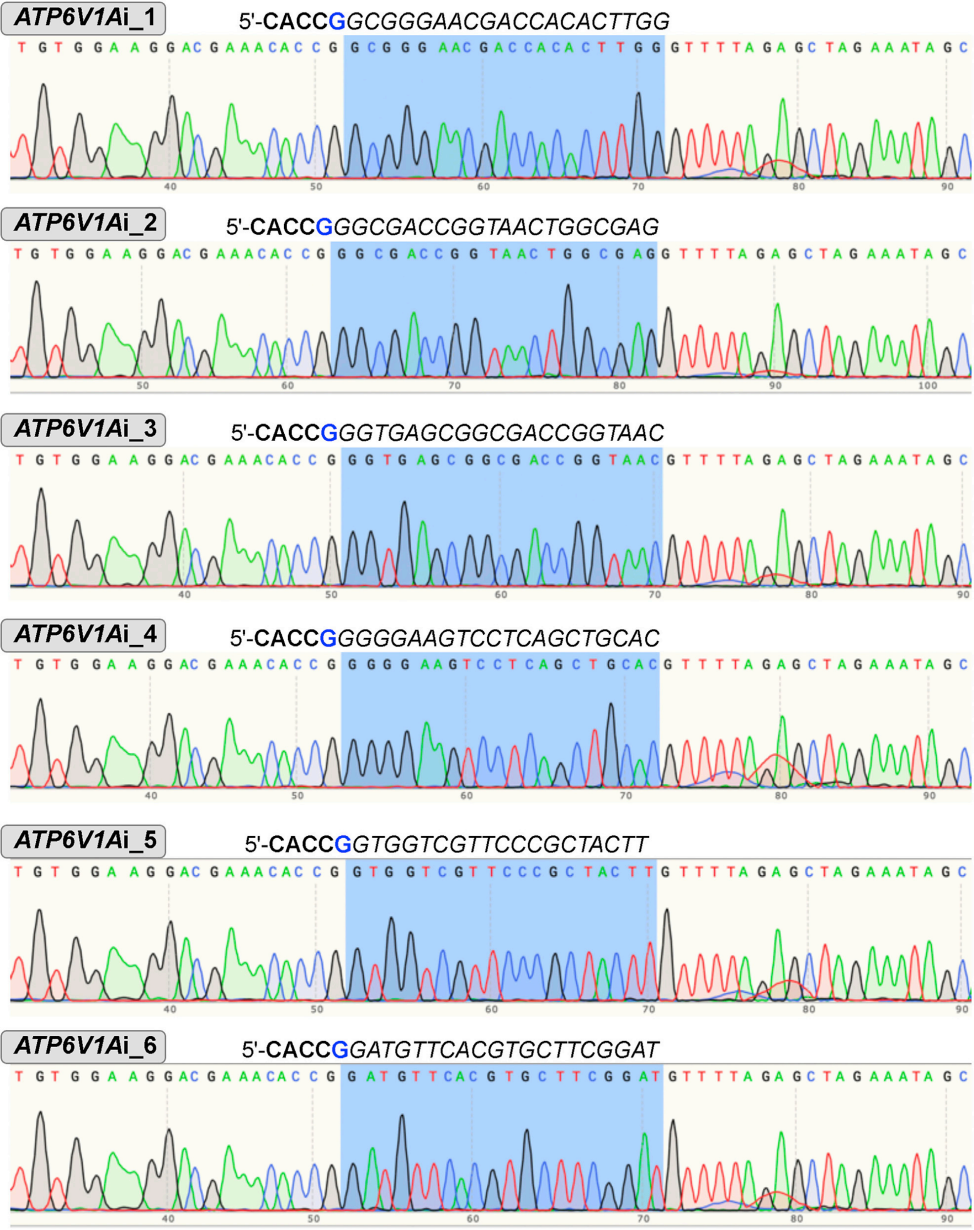
Sanger sequencing confirmation of six gRNA inserts in lentiGuide-Hygro-mTagBFP2, using the U6 primer, for *ATP6V1A* gene repression The flanking sequence from both sides of the insert illustrates the result of the cloning reaction.
  g. Prepare sufficient DNA (>12.2 μg) for lentivirus production in a 15-cm plate.

***Note:*** A single miniprep should provide enough DNA for a single virus prep; scale up to a Midi or maxiprep if more DNA is needed.

8. Validation of cloning by Sanger sequencing
  a. Select 6 individual colonies (not satellite colonies) from each LB plate.
  b. Put a single colony into 4 mL of TB medium with 100 μg/mL ampicillin.
  c. Incubate at 37°C, shaking at 300 rpm for approximately 16 h.
  d. Extract DNA using GeneJET Plasmid Miniprep Kit, or a miniprep kit from an alternative vendor, such as QIAprep Spin Miniprep Kit. Follow manufacturer's instructions.
  e. Submit samples for DNA sequencing using the U6 primer.
  f. Verify cloning by confirmation of the gRNA inserts (Figure 5).

**Pause point:** The sequence-verified plasmid DNA can be stored at –20°C until future use.

***Note:*** In the case of *ATP6V1A* gRNAs cloning, we notice that Sanger sequencing verified a high efficiency cloning into the plasmid backbone.

### Lentivirus preparations

**Timing: 1 week**

9. Lentivirus Production
  **Timing: ∼4 days**
  This step uses a PEI (Polysciences, #23966-2)-based 293T cell transfection method to produce and harvest lentivirus for downstream 2D cell culture infection protocols.
  **CRITICAL:** NIH guidelines require the maintenance of a Biological Safety Level 2 (BSL-2) facility for work involving lentivirus. BSL-2 labs require: (1) all personnel to wear lab coats and gloves, (2) all procedures that may produce infectious aerosols or splashes to be performed within a biological safety cabinet (BSC), (3) an autoclave, or alternative method of decontamination, to be available, (4) for the laboratory doors to be self-closing, and (5) for sink and eyewash stations to be readily available. For more info, please refer to this link: https://www.cdc.gov/training/quicklearns/biosafety/).
  **CRITICAL:** Steps of lentivirus collection and concentration should be performed in a BSC.
  a. Perform transfection when 293T cells are at approximately 80% confluency on a 15-cm plate.
  b. Thaw or prewarm PEI and Opti-MEM on a 37°C water or bead bath.
  c. In a 15 mL conical tube, combine PEI and DNA mixture in a 1:1 ratio.
    i. Drop PEI mixture into DNA mixture slowly to avoid precipitating the DNA (can be seen as white wisps).

**Table:** 

DNA mixture	PEI mixture
250 μL Opti-MEM	250 μL Opti-MEM
8.1 μg MDL	110 μL PEI
3.1 μg Rev	
4.1 μg VSVG	
12.2 μg *ATP6V1A*i_1
    ii. Gently agitate once between drops to minimize precipitated DNA.
    iii. Gently swirl 5 times after adding all the PEI.
    iv. Invert 5 times and gently vortex for 10 sec.
  d. Incubate the mixture for 15 min at room temperature (20°C–25°C) (the solution will become translucent).
  e. Drop ∼700 μL of the PEI/DNA mixture onto a 15-cm plate with ∼80% confluent 293T cells and put the plate into the incubator.
  f. After 6–8 h, perform a full media change (15 mL).
  g. After 48 h, harvest the first batch of viral media into a 50 mL conical tube or another sterile container.
  h. Add 15 mL of media to the plate and return the plate to the incubator.
  i. After 24 h, harvest the second batch of viral media into a 50 mL conical tube or another sterile container.
  j. Discard the plate in an appropriate manner. Treat the 293T cell plates with a 10% bleach solution for 10 min and aspirate the solution before discarding plates into the biohazard bin.
  k. Filter collected viral media from both batches by passing through a 0.22 μm pore size filter into a 50 mL conical tube or another sterile container.
    ***Note:*** VSVG is an envelope protein, acting as an empty backbone for lentiviral production. MDL and Rev are the 3^rd^ generation lentiviral packaging plasmids that are required for generating functional lentiviral particles.
    **Pause point:** Store filtered viral media on ice in a bucket at 4°C for less than 3 days.
    **CRITICAL:** Under microscope, 293T cells should give very high transfection efficiency (≥97%) for all gRNAs. This is a necessity for high-titer virus production (see troubleshooting 2).

10. Lentivirus Concentration
  **Timing: ∼4 h**
  This step produces concentrated lentivirus in tissue culture media for use in 2D cell culture infection protocols.
  ***Note:*** It is recommended to concentrate lentivirus on the day of the second viral media harvest to maximize virus yield. However, the viral media can also be stored for less than 3 days at 4°C for concentrating.
  a. Secure the centrifuge rotor into the ultracentrifuge (Beckman Coulter L-100XP Ultracentrifuge, or Optima XE-90 with 32 SW-Ti rotor) and pre-cool to 4°C.
  b. In the BSC, using sterile forceps, insert empty 38.5-mL polypropylene centrifuge tubes into the metal centrifuge tubes for the rotor being utilized.
  c. Cap the metal centrifuge tubes and pre-chill in 4°C for 15 min.
  d. Retrieve tubes and add 25–38 mL of filtered viral media (see above, Lentivirus Production, step 9-k) into polypropylene centrifuge tubes.
  e. Cap metal centrifuge tubes and carefully bring to ultracentrifuge.
  f. Centrifuge samples at 45,891 × *g* at 4°C for 2 h.
  g. After centrifugation, bring metal tubes back to the BSC.
  h. Carefully open the metal centrifuge tubes and use sterile forceps to remove the inserted polypropylene centrifuge tubes.
  i. Remove supernatant media by gently tilting the polypropylene centrifuge tubes and aspirating close to the top.
  j. Gently resuspend virus pellet (almost invisible) with DMEM or medium of choice.
    i. Avoid pipetting up and down during resuspension more than 10 times.
    ii. Volume depends on how much you want to concentrate the virus.
    iii. Resuspension with 500 μL should yield virus on the order of 10^6^ IU/mL.
  k. Pipette the resuspended virus into a sterile 15 mL conical tube or 1.5 mL microcentrifuge tube.
  l. Centrifuge at 3,000 × g for 5 min to pellet any residual cell debris. Aliquot supernatant into 0.5 mL microcentrifuge tube.
    **Pause point:** Store at –80°C until future use.
    **CRITICAL:** Minimize freeze-thaw cycles: a single freeze-thaw cycle can reduce infection efficiency by 50%.

11. Lentivirus Titration
  **Timing: ∼ 2 h**
  This step uses the abm’s qPCR lentivirus titration kit (Cat#: LV900) to assess the amount of virus particles in the concentrated lentiviral preparations. These steps follow those outlined in the manufacturer’s protocol.
  a. In a 0.5 mL microcentrifuge tube, add 2 μL of lentivirus to 18 μL of PBS (1:10 dilution).
  b. In a 0.5 mL microcentrifuge tube, add 2 μL of the diluted virus (from step 11-a) to 18 μL of Virus Lysis Buffer (1:10 dilution).
    i. Gently mix by swirling pipette tip around or tapping tube.
  c. Incubate mixture at room temperature (20°C–25°C) for 3 min. This solution is now referred to as the ‘viral lysate’.
    ***Note:*** The Ct value of the viral lysate will be used to determine the titer of the concentrated lentivirus preparation. The viral sample has since been diluted 1:10 twice; therefore, these dilution factors need to be considered in the titration calculation.
  d. Set-up qPCR reactions in triplicate, except negative control (NTC).

**Table:** 

Lentivirus qPCR reaction setup				
Component	Viral lysate	Positive control (STD1)	Positive control (STD2)	Negative control (NTC)
2× SYBR-Green	12.5 μL	12.5 μL	12.5 μL	12.5 μL
Viral lysate	2.5 μL	-	-	(2.5 μL DMEM)
Standard replicate 1 (STD1)	-	2.5 μL	-	-
Standard replicate 2 (STD2)	-	-	2.5 μL	-
Reagent-mix	10 μL	10 μL	10 μL	10 μL
Final volume per reaction	25 μL	25 μL	25 μL	25 μL
Total number of reactions	3	3	3	1
  e. Program the real-time qPCR instrument with the following cycle protocol (Figure 6).

**Table:** 

Lentivirus qPCR cycling conditions			
Steps	Temperature	Time	Cycles
Reverse Transcription	42°C	20 min	1
Enzyme Activation	95°C	10 min	1
Denaturation	95°C	15 s	40
Annealing/Extension	60°C	1 min

**Figure 6:**
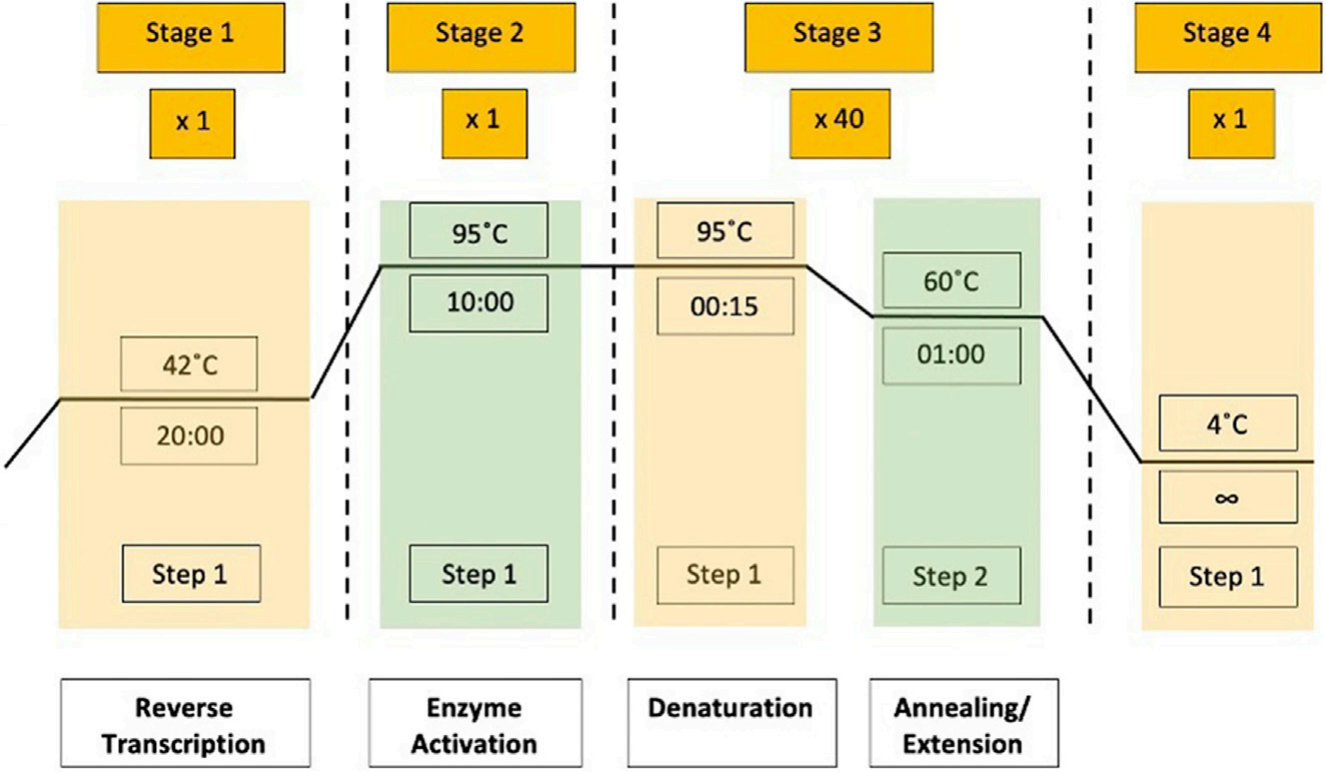
Thermocycler program for quantitative PCR
  f. Calculate the titer by using abm’s online calculator at http://www.abmgood.com/High-Titer-Lentivirus-Calculation.html. Alternatively, the following formulas can be used (see troubleshooting 3):

Titer of viral lysate = [**100 ×**] 5 × 10^7^/2^3(Ctx-Ct1)/(Ct2-Ct1)^

Ctx = Average of 3 Ct values of the unknown sample

Ct1 = Average of 3 Ct values of STD1

Ct2 = Average of 3 Ct values of STD2

***Note:*** Because the viral lysate had been diluted 1:10 twice, a total dilution factor of 100× was factored into the titer calculation. Adjust the dilution factor as needed.

### Lentiviral spinfection

**Timing: ∼6 h**

The time of spinfection and the G-force may increase the transduction efficiency.

12. Typically, 2.5–5.0 × 10^5^ NPCs are seeded into one well of a 24-well plate and evenly distributed. Cells are incubated overnight (18–24 h).
13. On day -1, exchange with NPC medium containing pLV-TetO-h*NGN2*-eGFP-P2A-Neo, FUW-M2rtTA, and CRISPRi-gRNA in lentiGuide-Hygro-mTagBFP2.

**Table:** 

Component (12-well)	Control	*ATP6V1A*i_1	*ATP6V1A*i_2
NPC medium (also for virus dilution)	860 μL	860 μL	860 μL
Virus of FUW-M2rtTA	5 μL	5 μL	5 μL
Virus of pLV-TetO-h*NGN2*-eGFP-P2A-Neo	10 μL	10 μL	10 μL
Virus of lentiGuide-Hygro-mTagBFP2	25 μL	-	-
Virus of *ATP6V1A*i_1	-	25 μL	-
Virus of *ATP6V1A*i_2	-	-	25 μL
Volume per well	900 μL	900 μL	900 μL
Differentiation replicates	3	3	3

14. Keep the plate in the incubator at 37°C for 15 min.
15. Place the plate in the plate centrifuge and spin at 1,000 × *g*, 25°C for 1 h.
16. Return the plate back to the 37°C incubator.
17. Replaced with fresh NPC medium after 3–4 h of transduction.

***Note:*** Purpose of each virus: FUW-M2rtTA activates the tetracycline activator to induce *NGN2* expression to initiate neuron differentiation. pLV-TetO-h*NGN2*-eGFP-P2A-Neo expresses human neurogenin-2 under control of TetON promoter, to generate *NGN2*-induced neurons from hiPSCs and hiPSC-NPCs. LentiGuide-Hygro-mTagBFP2 serves as lentiviral backbone for gRNA delivery to gene of interest. *ATP6V1A*i_1/2 contain synthetic gRNA for gene-targeted perturbation.

***Note:*** We need to pre-install Microscopy Filter Cubes for BFP (Excitation: 390 nm, Emission: 460 nm) (see troubleshooting 4) to check whether cells are blue-positive, determining the efficiency of lentiviral transduction. Next day, in spite of faint blue signal, there should be a >90% transduction efficiency across donors post infection.

### Neuronal differentiation

**Timing: 21 days**

**Figure 7:**
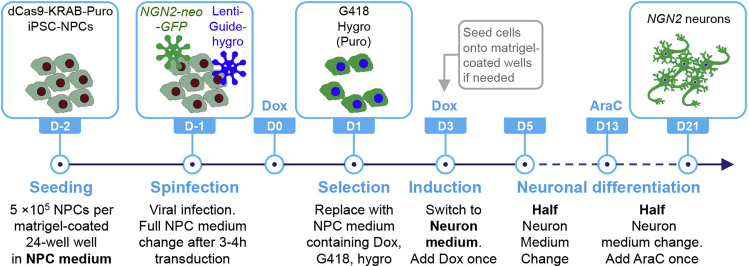
Scheme of *NGN*2-neuronal differentiation from human dCas9-KRAB hiPSC-derived NPCs

Exogenous human *NGN2* transduction can rapidly induce NPCs into functional glutamatergic neurons in three weeks. See the schematic overview of our strategy for neuronal differentiation in our study (Figure 7).

18. Doxycycline (Dox) induction, hygromycin (hygro) and neomycin (G418) selection

**Table:** 

Antibiotics	Resistance gene in plasmids	Selection	Final concentration	Time
Hygromycin	lentiGuide-Hygro-mTagBFP2	gRNA-infected	1 mg/mL	Day 1–3
G418 (Geneticin)	pLV-TetO-h*NGN2*-eGFP-Neo	*NGN2*-infected	1 mg/mL	Day 1–3
Puromycin	lenti-EF1a-dCas9-KRAB-Puro	dCas9-KRAB NPCs	1 μg/mL	Day 1–3

  a. On day 0, replaced with fresh NPC medium containing Dox (1 μg/mL).
  b. On day 1, check GFP and BFP signals under epifluorescence microscope (usually >90% efficiencies). Replaced with fresh NPC medium containing Dox, hygro, and G418 (optionally with puromycin if concerned with the loss of dCas9-KRAB transgene in NPCs) (see troubleshooting 5).
  c. On day 3, a large number of cells may die due to antibiotic selection. Replace with neuron medium (see materials and equipment) containing Dox (1 μg/mL).

19. During day 5–13, half media change every 2 days.
20. During day 13–21, a complete media change to remove Dox and add 50 nM Cytosineb-D- arabinofuranoside hydrochloride (Ara-C) into neuron medium.
  Ara-C reduces the proliferation of non-neuronal cells in the culture.
21. Gently replace half of the media every other day. Add media towards the wall of the well to avoid neuron detachment (see troubleshooting 6).
22. On day 21 (D21), fix D21 *NGN2* neurons with 4% paraformaldehyde (PFA) and characterize them by immunofluorescence with TUJ1 (1:1,000, #801202, mouse IgG2a) and MAP2 (1:2,000, ab5392, chicken antibody).

**Pause point:** D21 *NGN2* neurons can be fixed with 4% PFA and stored at 4°C for future immunofluorescence. TRIzol/RIPA-lysed samples can be stored at –20°C for future biochemical assays.

***Note:*** Dox induction should yield a high percentage of GFP-positive cells.

***Note:*** To measure the neuronal electric activity, we strongly recommend *NGN2* neurons should be co-cultured with human fetal astrocytes to enhance neuronal maturation. On day 3, astrocytes are split as 17,000 cells/well in a Matrigel-coated 48W MEA plate (Axion Biosystems, M768-tMEA-48W) and maintained as above. On day 7, 70,000 *NGN2* neurons are detached, spun down, and seeded on the astrocytes culture. We can detect the population-wide neuronal activity, including frequency and coordination of network firing, and the amplitude of voltage-gated potassium (K^+^) current and sodium (Na^+^) current using an Axion multi-electrode array (MEA) or whole-cell patch-clamp recordings respectively. We can keep the neuronal culture up to 35 days for recording if necessary.

**CRITICAL:** If there is a need to seed cells on to a microplate, such as a CytoView 48-well MEA plate, for neuronal activity measurement, then we highly recommend that you split *NGN2* neurons using Accutase before differentiation day 7. Dissociating neurons at later stages of neuronal differentiation may lead to increased cell death and potential loss of cultures.

### Validation of CRISPRi-induced gene repression by qPCR and western blot

**Timing: 2 days**

The validation step is a critical prerequisite before starting a functional study. There is a necessity to redesign and test new gRNAs if CRISPRi fails to significantly repress gene mRNA and protein expression (see troubleshooting 7). Reduced gene expression may vary greatly according to genes, gRNAs, transduced tissue types, and batches. We expect a consistent and significant reduction of gene transcription and protein translation. A satisfying level of CRISPRi can sufficiently knockdown the genes of interest to yield a phenotype, even though a small reduction may be achieved.

23. Quantitative PCR validation
  a. RNA extraction: 21-day *NGN2* neurons in 250 μL of TRIzol Reagent (Invitrogen, #10296028) per 24-well well. Add 50 μL of Chloroform (Fisher Chemical, #C298). Spin in a bench-top centrifuge (Eppendorf Centrifuge 5702R, or Thermo Scientific Heraeus Multifuge X1R Refrigerated Benchtop Centrifuge), at maximum speed (21,130 × *g*) at 4°C for 15 min. Transfer the clear aqueous phase to tubes with 150 μL of isopropanol (Fisher Bioreagents, #BP2618) and 1 μL of GlycoBlue (Thermo Fisher Scientific, #AM9516) or Glycogen (Thermo Fisher Scientific, #R0551), taking care not to carry over the white interface. Spin at maximum speed (21,130 × *g*) at 4°C for 15 min. Wash the RNA with ice cold 70% ethanol (Fisher Bioreagents, #BP201851). Resuspend in 50 μL of UltraPure DNase/RNase-free distilled water (Invitrogen, #10977) and adjust concentration to 25 ng/μL.
  b. Follow manufacturer's instructions of TaqMan RNA-to-Ct 1-Step Kit (Invitrogen, #4392938). Alternatively, Reverse transcribe RNA into cDNA using the Promega RT system (Promega, #A3500). Run samples on the Applied Biosystems StepOnePlus Real-Time PCR machine using SYBR Select Master Mix (Applied Biosystems, #4472903). Design qPCR primers for genes of interest of the users. In our study, primers for *ATP6V1A* and the housekeeping gene of β-*Actin* are listed below.

**Table:** 

Gene_id	Primer_FWD	Primer_REV	Length
*ATP6V1A*	GAGATCCTGTACTTCGCACTG	GGGATGTAGATGCTTTGGGTC	130
β*-Actin*	TGTCCCCCAACTTGAGATGT	TGTGCACTTTTATTCAACTGGTC	109
  c. One-step real-time RT-PCR is quicker to set up and involves less RNA handling. Combine the following in a PCR tube for gene expression assay.

**Table:** 

Reagent	Amount	Master Mix
2× Power SYBR Green RT-PCR Mix	5 μL	× N
125× RT Enzyme Mix	0.08 μL	× N
DNase/RNase-free distilled water	2.72 μL	× N
FWD Primer + REV Primer (mix)	0.2 μL	× N
**Master Mix Total**	8 μL	8 × N
25 ng/μL RNA	2 μL	
**Reaction Total**	10 μL
  d. Set up triplicates and run the QuantStudio 7 Flex Real-Time PCR.

**Table:** 

PCR Cycling Conditions			
Steps	Temp.	Time	Cycles
Hold Stage	48°C	30 min	1
Hold Stage	95°C	10 min	1
PCR Stage	95°C	15 s	40
	60°C	1 min	
Melt Curve Stage	95°C	15 s	
	60°C	1 min
  e. Calculate the value of 2ˆ-ΔΔCt to get the expression fold change by comparing CRISPRi-mediated neurons to the control with empty lentiGuide-Hygro-mTagBFP2 or a scrambled plasmid.
    **CRITICAL:** Always use TRIzol and chloroform in a functioning fume hood.

24 Western blot validation if commercial antibody is available.
  a. Protein sample preparation: 21-day *NGN2* neurons are rinsed with ice-cold phosphate-buffered saline (PBS), pelleted, and lysed in RIPA Lysis and Extraction Buffer (100 μL/12-well well, Thermo Fisher Scientific, #89900) containing Halt Protease and Phosphatase Inhibitor Cocktail (1×, Thermo Fisher Scientific, #78440). Alternatively, we can make our own RIPA lysis solution (see materials and equipment).
  b. Samples are sonicated for 1 min then centrifuged at 15,871 × *g* for 10 min.
  c. The supernatant is collected, and the total protein concentration is determined by using Quick Start Bradford Protein Assay (Bio-Rad, #5000201) following the manufacturer’s instructions.
  d. Use an antibody (ATP6V1A rabbit antibody: ab199326, 1:1,000 dilution) to obtain the protein expression of the target protein. β-Actin (mouse monoclonal antibody, ab8227, 1:1,000 dilution) is used as a loading control.
  e. Secondary antibodies used include IRDye 800CW Donkey anti-Mouse IgG (1:15,000, LI-COR, #925-32212) and IRDye 680RD Donkey anti-Rabbit IgG (1:15,000, LI-COR, #925-68073).
  f. Capture and quantify images using the Odyssey Imaging Systems (LI-COR Inc.). Alternatively, we can apply a conventional western blot using enhanced chemiluminescent (ECL, GE, #RPN2236).

***Note:*** Antibody for the detection of novel target proteins is not always available.

## Expected outcomes

**Figure 8:**
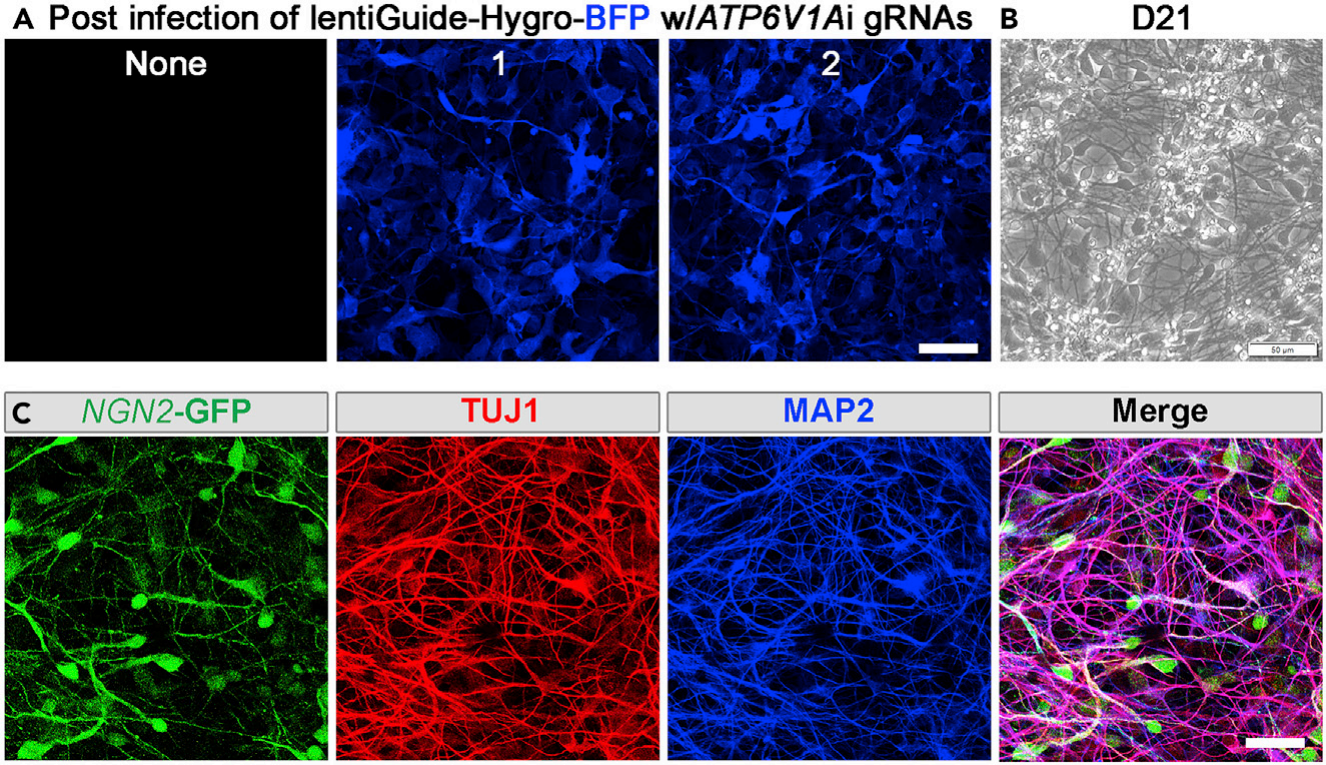
Characterization of virus infection and *NGN*2-neuronal differentiation (A) BFP expression (Blue) of gRNA candidates 1 and 2 post virus infection. (B) Representative bright-field image of D21 *NGN2* neurons. (C) Dox-induced D21 *NGN2* neurons (GFP: green) express TUJ1 (red) and MAP2 (blue), pan-neuronal markers. Bars, 50 μm: A–C.

The lentiGuide viral delivery has a robust transduction result of >90% BFP-positive cells after 48 h post-infection (Figure 8A). The BFP signal should remain constant throughout neuronal maturation. On differentiation day 21, NPC-induced glutamatergic neurons show neuronal cell morphology with multifold neurite extensions (Figure 8B). After Dox withdrawal the *NGN2*-GFP signal may gradually decrease. Cells are immunostained with MAP2 and TUJ1 (Figure 8C), and images are captured using a confocal microscope (LSM 780, Zeiss) with a 63× objective lens. We expect to generate a highly homogeneous neuronal population.

**Figure 9:**
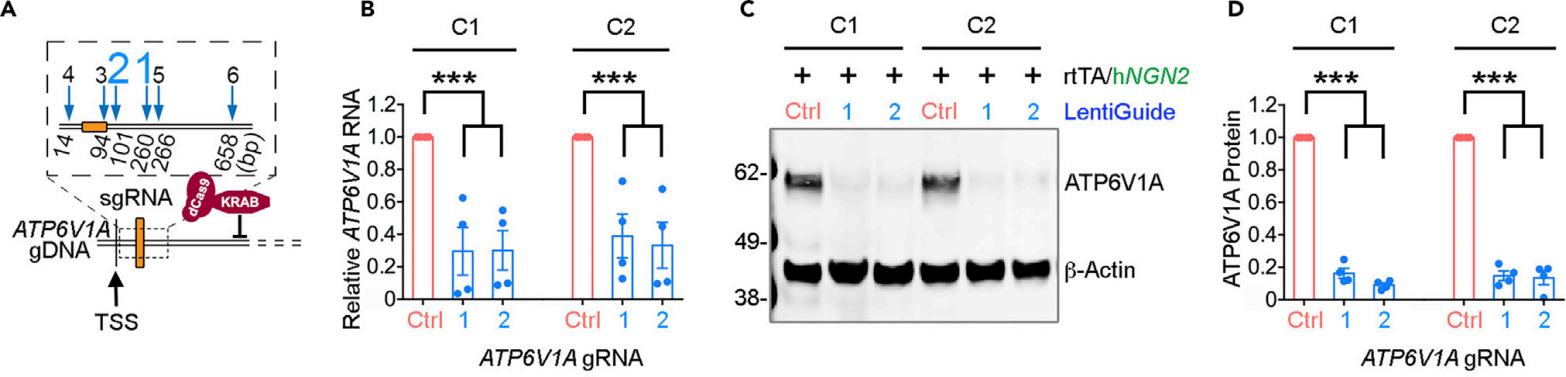
*ATP6V1A* RNA and protein levels reduced significantly in *NGN*2 neurons post gRNA perturbation (A and B) qPCR analysis (n = 4) confirms the decreased *ATP6V1A* RNA by gRNA candidates 1 and 2 in *NGN2* neurons of two independent donors (i.e., C1 and C2). (C and D) Representative western blot and quantitative analysis (n = 4) of ATP6V1A protein levels in *NGN2* neurons. β-Actin is a loading control. ANOVA with Dunnett’s test; ∗∗∗p < 0.001.

In our study, we designed six gRNAs to target the promoter region for knockdown (KD) of *ATP6V1A* (Figure 9A) and identified two gRNAs (*ATP6V1A*i_1 and i_2) that efficiently repressed *ATP6V1A* in dCas9-KRAB NPC-derived *NGN2* neurons. The *ATP6V1A* RNA (60%–70% repression, p < 0.001; Figure 9B) and protein levels (80%–90% repression, p < 0.001; Figures 9C and 9D) were reduced significantly. RNA-seq analysis (GEO: GSE128367) of D21 *NGN2* neurons (n = 20 samples) also indicated two gRNAs significantly reduced *ATP6V1A* RNA expression.

## Limitations

CRISPRi provides a powerful tool to decipher the impact of down-regulation of gene expression in development and disease. One must carefully design knockdown experiments to distinguish the impact of CRISPRi on neuronal patterning, maturation, and function; towards this, the dCas9-KRAB effector and/or gRNAs can be delivered or induced to express either early or late in experimental design. We find that a minimum of seven days is necessary to ensure robust knockdown.

The approach depends on a successful search for effective gRNAs. It is critical to validate each gRNA in every donor and cell type, as the magnitude of CRISPRi achieved varies significantly according to cellular context. On occasion, we have failed to detect any gRNA that significantly impacts the expression of a gene of interest (Ho et al., 2017). For single-cell experiments, particularly pooled CRISPR screening experiments (Tian et al., 2019), it may be necessary to ensure a comparable degree of knockdown between all neurons in the population, which is likely to be best achieved by generating clonal CRISPRi hiPSCs that express dCas9-KRAB equally in all cells.

It is critical to rule out potential cis- and trans- off-target effects, which may arise either from homologous sequence or proximity in the linear (Schrode et al., 2019) or 3D genome. For this reason, CRISPRi may not be ideal if: (**a**), the targeting sequence is a bidirectional promoter, or (**b**), the transcripts for the gene of interest overlap or are very close to the transcripts of one or more different genes.

In theory, the KRAB domain transcriptionally represses all RNA isoforms of a gene of interest. For this, CasRx approaches that bind RNA targets may be a more suitable approach for the functional evaluation of the impact of down-regulation of specific isoforms or RNA splicing.

## Troubleshooting

### Problem 1

No colonies grow on agar plates after transformation (step 7-j).

### Potential solution

The quantity and quality of DNA is not sufficient for plasmid transformation.

Solution 1: Increase the amount of DNA when mixed with NEB 10-beta competent cells.

Solution 2: There might be a need to re-do the Golden Gate Assembly. Pay more attention when pipetting small volumes.

Solution 3: Check the sgRNA design to make sure gRNAs can be ligated into the BsmBI-digested lentiGuide-Hygro-mTagBFP2.

### Problem 2

BFP fluorescence is low at the 36h point after initial viral transfection of 293T cells (step 9-CRITICAL).

### Potential solution

We have found that replacing PEI with PEIMax (Polysciences #24765; 1 MG/ML pH 7.0 stock) at a ratio of 1 μg DNA to 1 μL PEIMax can significantly increase the transfection efficiency during lentiviral production. To optimize this protocol with new DNA and PEIMax stocks, you will need to test transfection efficiency ranging from 1:1 to 1:10 and pick the concentration with the highest efficiency.

### Problem 3

Lentiviral titer is low after virus concentration by ultracentrifuge (step 11-f).

### Potential solution

We found this problem to be most likely associated with the low transfection of 293T cells. It is better to optimize the protocol and plasmid concentrations to improve the transfection efficiency.

### Problem 4

gRNA BFP signal cannot be detected in transduced NPCs/neurons using fluorescent microscopy (step 17-Note).

### Potential solution

We should exclude the possibility of inefficient gRNA transduction. Transduction efficiency may increase by the infection of suspension cells after seeding.

BFP signal usually is faint in the days immediately following transduction and may be difficult to determine by eye. The strength of the signal should increase until it peaks around 3–4 days after transduction. We advise keeping a control well that has the gRNA vector but has not been transduced; this control will act as a comparison to the cultures with and without BFP signal.

### Problem 5

Cultures perish during selection with multiple antibiotics (step 18-b).

### Potential solution

Antibiotic selection may be staggered (i.e., G418 then hygro selection) to aid culture viability. Typically, problems with culture viability during antibiotic selection arise from low viral transduction rates. Check GFP and BFP signals before selection with antibiotics to ensure viral transduction is successful.

### Problem 6

During neuronal differentiation, *NGN2* neuronal cultures clump together, risking detachment and interfering with phenotyping of mature cultures (step 21).

### Potential solution

This is typically an issue with Matrigel coating. A strong recommendation is to optimize Matrigel concentration prior to start an extensive experiment. Sequential coating with polyethylenimine (PEI, branched) and Matrigel coating promotes firm attachment. This coating method is well suited for imaging purpose.

Clumping may be reduced during seeding by gently pipetting NPC pellets after spinning using a P1000 tip to break apart cell clusters and aid resuspension of cells as a monolayer.

### Problem 7

Low efficiency gene repression (steps 23 and 24).

### Potential solution

Insufficient gene repression may occur for several reasons. We need to:

• Increase the viral amount of gRNA, if the problem is due to a low lentiviral transduction.
• Check whether low expression of specific genes may be causing apoptotic cell death. The cells with low gene expression will be washed away when changing the media.
• Measure dCas9 expression levels in parental NPCs or *NGN2* neurons.
• Combine multiple gRNAs that may lead to significant repression. If the problem continues, design and test more gRNAs.

## Resource availability

### Lead contact

Stable hiPSC-derived neuronal progenitor cells (hiPSC-NPCs) expressing dCas9-KRAB and *ATP6V1A*i-gRNA in lentiGuide-Hygro-mTagBFP2 utilized in this study can be requested through Kristen Brennand (kristen.brennand@mssm.edu) upon Material Transfer Agreement.

More information and requests for resources and reagents should be directed to and will be fulfilled by the lead contact, Aiqun Li (aiqun.li@mssm.edu).

### Materials availability

This study did not generate any unique reagents.

### Data and code availability

The accession number for *ATP6V1A* knockdown RNA sequencing data reported in this paper is Gene expression omnibus (GEO): GSE128367.
